# Supplementary material for: Unveiling Trail Making Test: visual and manual trajectories indexing multiple executive processes
Source: Sci Rep. 2022 Aug 22;12:14265. doi: 10.1038/s41598-022-16431-9 (PMC9395513; doi:10.1038/s41598-022-16431-9)
Supplement: Supplementary file 1 — Supplementary Information. [file 41598_2022_16431_MOESM1_ESM.pdf]

# Unveiling Trail Making Test: Visual and manual trajectories indexing multiple executive processes

Linari, Ignacio[1*]; Juantorena, Gustavo E[1*]; Ibáñez, Agustín[2,3,4,5]; Petroni, Agustín[1,6^];

Kamienkowski, Juan E[1,7^+]

[1] Laboratorio de Inteligencia Artificial Aplicada, Instituto de Ciencias de la Computación, Facultad de Ciencias Exactas y Naturales, Universidad de Buenos Aires - CONICET, Argentina

[2] Cognitive Neuroscience Center (CNC), Universidad de San Andrés, and National Scientific and Technical Research Council (CONICET), Buenos Aires, Argentina

[3] Global Brain Health Institute (GBHI), University of California San Francisco (UCSF), San Francisco, US

[4] Trinity College Dublin (TCD), Ireland

[5] Latin American Brain Health Institute (BrainLat), Universidad Adolfo Ibáñez, Santiago, Chile

[6] University of Gothenburg, Sweden

[7] Maestría de Explotación de Datos y Descubrimiento del Conocimiento, Facultad de Ciencias Exactas y Naturales, Universidad de Buenos Aires, Argentina

*,^ Equal contributions

+ Corresponding author:

Juan Esteban Kamienkowski

Departamento de Computación, Facultad de Ciencias Exactas y Naturales, Universidad de Buenos Aires

Pabellón 1, Ciudad Universitaria

(1428) Ciudad Autónoma de Buenos Aires

Argentina

e-mail: juank@dc.uba.ar; jkamienk@gmail.com

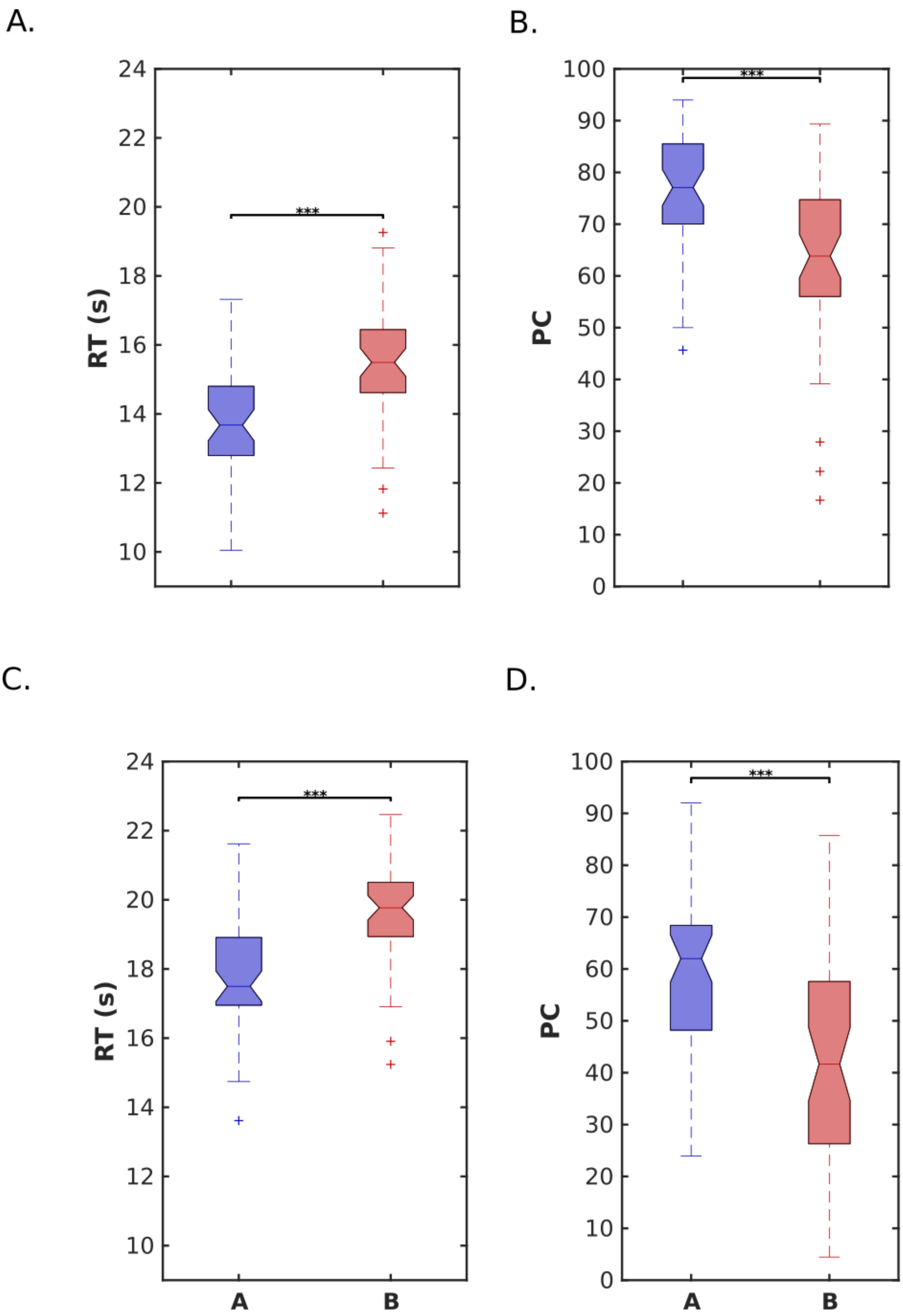


**Supplementary Figure 1.** Performance measures for two different target thresholds. For a threshold of 10 targets: **A.** RT: Wilcoxon signed-rank test: $p=1.1*10^{-9}$, z=-6.1, e.s.=0.87 **B.** PC: Wilcoxon signed-rank test: $p=1.1*10^{-9}$, z=6.1, e.s.=0.87. For a threshold of 14 targets **C.** RT: Wilcoxon signed-rank test: $p=1.1*10^{-9}$, z=-6.1, e.s.=0.87 **D.** PC: Wilcoxon signed-rank test: $p=1.1*10^{-9}$, z=6, e.s.=0.87.

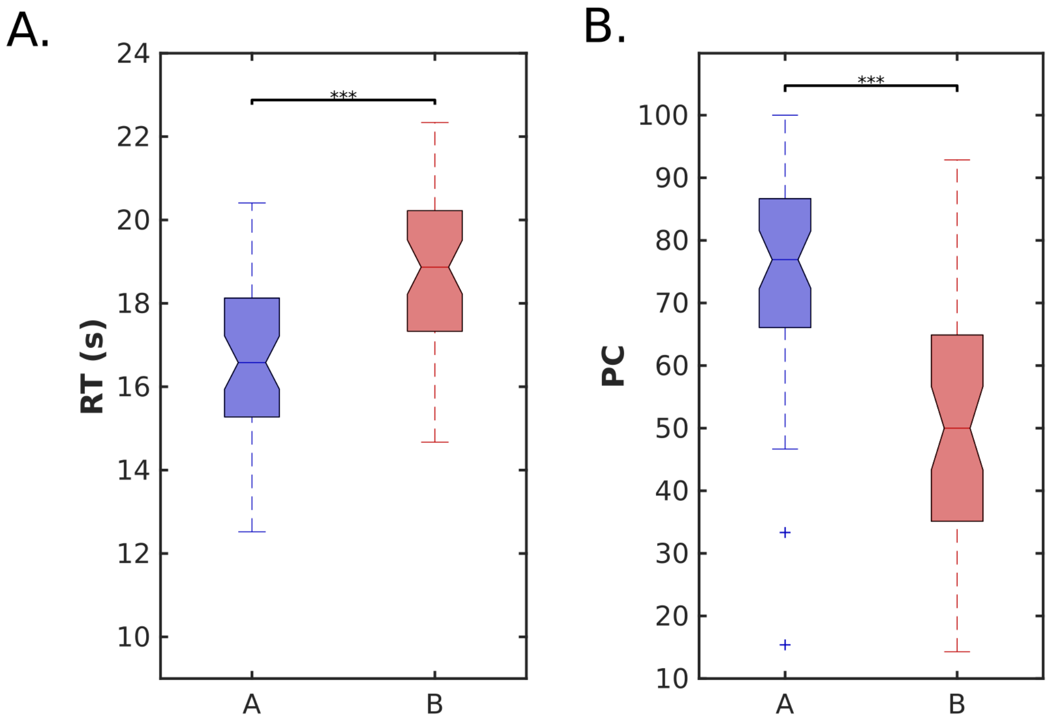


**Supplementary Figure 2.** Performance measures for the first ⅓ trials of the test (15A and 15B) discarding the very first 2 trials due to starting errors (trials 3:32). **A.** RT: Wilcoxon signed-rank test: $p=9.7*10^{-9}$, z=-5.7, e.s.=0.81 **B.** PC: Wilcoxon signed-rank test: $p=1.8*10^{-8}$, z=5.6, e.s.=0.8.

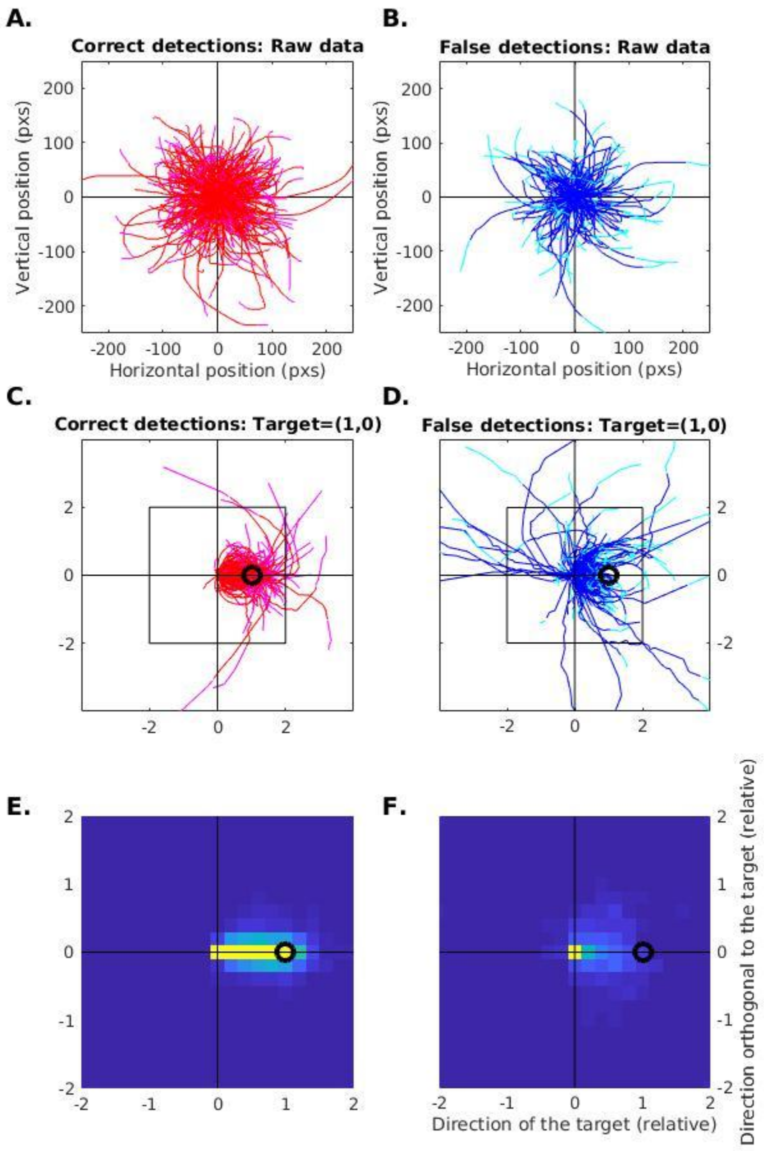


**Supplementary Figure 3.** One participant. Spatial distribution of the paths explored by the hand when fixating a new item, for **A.** Correct detections (raw data), **B.** False detections (raw data), **C.** Correct detections (aligned so that the fixated item was at (0,1)), **D.** False detections (aligned so that the fixated item was at (0,1)), **E.** aligned and normalised for Correct detections and **F.** aligned and normalised for False detections.

**TMT example of the phase classification**

**Supplementary Video.** Segmentation of one trial (TMT-B). The dots represent the fixations coloured by the segmentation of the task: Red: Planning, Green: Exploratory, Blue: Monitoring. And the trajectory of the mouse is presented in Grey. The video is played in Real-Time.
